# German evidence- and consensus-based guidelines 2011 for the treatment of juvenile idiopathic arthritis (JIA)

**DOI:** 10.1186/1546-0096-10-S1-A53

**Published:** 2012-07-13

**Authors:** Gregor Dueckers, Nihal Guellac, Martin Arbogast, Guenther Dannecker, Ivan Foeldvari, Michael Frosch, Gerd Ganser, Arnd Heiligenhaus, Gerd Horneff, Arnold Illhardt, Ruediger Krauspe, Matthias Schneider, Wolfram Singendonk, Helmut Sitter, Barbara Markus, Marianne Spamer, Norbert Wagner, Tim Niehues

**Affiliations:** 1 Asklepios Kinderklinik, St. Augustin, NRW, Germany; 2 Berufsverband, Berlin, Germany; 3 Deutsche Rheuma Liga E.V., Bonn, NRW, Germany; 4 Deutsches Zentrum für Kinder- und Jugendrheumatologie, Garmisch-Partenkirchen, Bayern, Germany; 5 HELIOS Klinikum, Krefeld, NRW, Germany; 6 Klinikum Eilbeck, Hamburg, HH, Germany; 7 Olgahospital Stuttgart, Stuttgart, BW, Germany; 8 Rheumazentrum Oberammergau, Oberammergau, Bayern, Germany; 9 RWTH Universitaetsklinikum, Aachen, NRW, Germany; 10 St. Franziskus Hospital, Münster, NRW, Germany; 11 St. Josef Stift, Sendenhorst, NRW, Germany; 12 Universitaet Duesseldorf, Duesseldorf, NRW, Germany; 13 Universitaet Marburg, Marburg, Hessen, Germany; 14 Universitaet Muenster, Muenster, NRW, Germany

## Purpose

Standardisation of care opens the chance to further improve the quality of care for children and adolescents with JIA. We aimed to update our interdisciplinary, evidence-based clinical practice guidelines for the treatment of JIA. Our update is based on the existing German guidelines of 1999^*^ and 2005^*^ and 2008^#^ (^*^published as book chapters; ^#^peer review publication in Clinical Research and Practice in Pediatrics 2008; 220: 392 - 402).

## Methods

We performed a systematic literature analysis (deadline: 15^th^ January 2010) in PUBMED with the key words “juvenile idiopathic (rheumatoid) arthritis” and “therapy”. As limits in PUBMED we used: humans, published in the last 3 years, all child 0-18 years, clinical trial. Studies were evaluated for quality of methodology. Studies relating to diagnosis of JIA, uveitis, vaccination, transition and rofexocibe were excluded. Authors of the 2005 guideline and representatives attended consensus conferences, held on 9^th^ of may 2007, 1^st^ of August 2007 and 15^th^ of January 2010 at Düsseldorf respectively Krefeld, Germany. Conferences were hosted by a professional moderator and were attended by 95 % of the representatives who had been named by interdisciplinary scientific societies. Scientific societies and organisations represented paediatricians in practice and hopistals, adult and pediatric rheumatologists, orthopaedics, ophthalmologists, surgeons, physiotherapists, national and local support-groups for parents and children. We edited a manuscript with core conclusions of the studies. Statements were discussed and confirmed in a Delphi method.

## Results

The 2010 version of guideline inlcudes 15 statements. Updated consensus statements and key notes regarding drug therapy, symptomatic and surgical management of JIA were compiled and judged strictly by the criteria of Evidence-Based Medicine (EBM).

## Conclusion

Many interventions in the treatment of JIA can now be based on high level evidence as the number of randomized controlled clinical trials is increasing: In a first step it is recommended that JIA is treated with NSAR followed by IATH and/or MTX. Other interventions such as the role of biological agents, physiotherapy, arthroscopy, etc. are discussed strictly on the basis of literature available. Complementing these data with the long-standing experience of caregivers allows to create recommendations that may improve the quality of care for children and adolescents with JIA.

## Disclosure

Gregor Dueckers: Baxter, 9, Novartis Pharmaceuticals Corporation, 9; Nihal Guellac: None; Martin Arbogast: None; Guenther Dannecker: None; Ivan Foeldvari: Abbott Laboratories, 9, Chugai, 9, Pfizer Inc, 9; Michael Frosch: None; Gerd Ganser: None; Arnd Heiligenhaus: Abbott Immunology Pharmaceuticals, 2, Alcon, 2, Novartis Pharmaceuticals Corporation, 2; Gerd Horneff: Abbott Immunology Pharmaceuticals, 2, 5, 6, Bristol-Myers Squibb, 5, Chugai, 5, 6, Nycomed, 5, 6, Pfizer Inc, 2, 5, 6, 8, Sandoz, 5, 6; Arnold Illhardt: None; Ruediger Krauspe: None; Matthias Schneider: None; Wolfram Singendonk: None; Helmut Sitter: None; Barbara Markus: None; Marianne Spamer: None; Norbert Wagner: None; Tim Niehues: Abbott Immunology Pharmaceuticals, 5, Essex Pharma, 5, Novartis Pharmaceuticals Corporation, 5, Pfizer Inc, 5, Wyeth Pharmaceuticals, 5.

